# Quantitative miRNA Expression Analysis Using Fluidigm Microfluidics Dynamic Arrays

**DOI:** 10.1186/1471-2164-12-144

**Published:** 2011-03-09

**Authors:** Jin Sung Jang, Vernadette A Simon, Rod M Feddersen, Fariborz Rakhshan, Debra A Schultz, Michael A Zschunke, Wilma L Lingle, Christopher P Kolbert, Jin Jen

**Affiliations:** 1 Department of Pulmonary and Critical Care Medicine, 200 First Street SW, Rochester MN 55905, USA; 2 Gene Expression Shared Resource, Advanced Genomics Technology Center and the Mayo Clinic Cancer Center, 200 First Street SW, Rochester MN 55905, USA; 3 Department of Experimental Pathology, 200 First Street SW, Rochester MN 55905, USA

## Abstract

**Background:**

MicroRNAs (miRNAs) represent a growing class of small non-coding RNAs that are important regulators of gene expression in both plants and animals. Studies have shown that miRNAs play a critical role in human cancer and they can influence the level of cell proliferation and apoptosis by modulating gene expression. Currently, methods for the detection and measurement of miRNA expression include small and moderate-throughput technologies, such as standard quantitative PCR and microarray based analysis. However, these methods have several limitations when used in large clinical studies where a high-throughput and highly quantitative technology needed for the efficient characterization of a large number of miRNA transcripts in clinical samples. Furthermore, archival formalin fixed, paraffin embedded (FFPE) samples are increasingly becoming the primary resource for gene expression studies because fresh frozen (FF) samples are often difficult to obtain and requires special storage conditions. In this study, we evaluated the miRNA expression levels in FFPE and FF samples as well as several lung cancer cell lines employing a high throughput qPCR-based microfluidic technology. The results were compared to standard qPCR and hybridization-based microarray platforms using the same samples.

**Results:**

We demonstrated highly correlated Ct values between multiplex and singleplex RT reactions in standard qPCR assays for miRNA expression using total RNA from A549 (R = 0.98; p < 0.0001) and H1299 (R = 0.95; p < 0.0001) lung cancer cell lines. The Ct values generated by the microfluidic technology (Fluidigm 48.48 dynamic array systems) resulted in a left-shift toward lower Ct values compared to those observed by ABI 7900 HT (mean difference, 3.79), suggesting that the microfluidic technology exhibited a greater sensitivity. In addition, we show that as little as 10 ng total RNA can be used to reliably detect all 48 or 96 tested miRNAs using a 96-multiplexing RT reaction in both FFPE and FF samples. Finally, we compared miRNA expression measurements in both FFPE and FF samples by qPCR using the 96.96 dynamic array and Affymetrix microarrays. Fold change comparisons for comparable genes between the two platforms indicated that the overall correlation was R = 0.60. The maximum fold change detected by the Affymetrix microarray was 3.5 compared to 13 by the 96.96 dynamic array.

**Conclusion:**

The qPCR-array based microfluidic dynamic array platform can be used in conjunction with multiplexed RT reactions for miRNA gene expression profiling. We showed that this approach is highly reproducible and the results correlate closely with the existing singleplex qPCR platform at a throughput that is 5 to 20 times higher and a sample and reagent usage that was approximately 50-100 times lower than conventional assays. We established optimal conditions for using the Fluidigm microfluidic technology for rapid, cost effective, and customizable arrays for miRNA expression profiling and validation.

## Background

MicroRNAs (miRNAs) are short, single-stranded, noncoding RNAs that regulate gene expression by interacting with or inhibiting mRNA in both plants and animals [1-3]. To date, more than 800 human miRNAs have been identified and the total number is still increasing [4]. It is estimated that about two thirds of all protein-coding genes are regulated by miRNAs [5]. Although some miRNAs are yet to be characterized, biochemical and genetic studies have indicated that miRNA regulation is essential for biological processes such as development, differentiation, cell proliferation, and apoptosis [6-9]. Recent studies have demonstrated that miRNA genes can be aberrantly expressed in human cancers and they function as either oncogenes or tumor suppressor genes via regulation of target transcripts [10,11].

Although formalin fixed, paraffin embedded (FFPE) tissue samples typically contain fragmented nucleic acids, they are the most commonly available clinical specimens for histology and pathological analysis and are a critical resource for developing new molecular markers in the cancer research [12,13]. Because of their small size, miRNA molecules appear to be less prone to degradation, in contrast to mRNA expression studies, and no significant differences in miRNA expression between FFPE and FF samples have been observed [14-16].

Real-time quantitative PCR (qPCR) is considered a 'gold standard' for quantification of gene expression and has been widely employed as a validation method for microarray studies. However, the qPCR method is a relatively low throughput, high cost, and tedious technique typically performed in a 96 or 384 well plate format. The Fluidigm microfluidic technology uses the integrated fluidic circuits (IFC) which contain tens of thousands of microfluidic controlled valves and interconnected channels to move molecules of biological samples and reagents in a variety of patterns [17]. IFCs reduce a qPCR reaction from the routine 10-20 microliter volume down to a 10 nanoliter scale making it possible to perform routine qPCR analysis for thousands of reactions in a single run. This technology has been used for gene expression, genotyping, mutation detection, and absolute quantization of nucleic-acid sequences [17,18]. Spurgeon et al. [19] showed that microfluidic dynamic arrays can be used to simultaneously measure 48 mRNAs in several tissues. Wang et al. [20] developed a high throughput SNP genotyping assay demonstrating high accuracy and call rate in human samples using a nanofluidic platform. This new real-time PCR technology makes it possible to perform validation as well as high throughput gene expression measurements using very limited sample and reagent quantities [17,19].

Here we show that miRNA expression profiling and validation are possible using high throughput real-time quantitative PCR (qPCR) method with the microfluidic technology. FFPE and FF samples appear to perform similarly in this platform when cDNA are generated from 96 multiplexed RT reactions. We compared this new approach to the standard microarray-based technology. To our knowledge, this study is the first report comparing miRNA expression profiling between microarrays and qPCR using microfluidic arrays.

## Results

### Correlation of Reverse Transcription Efficiency between Single- and Multiplexed Primer Sets

**Figure 1:**
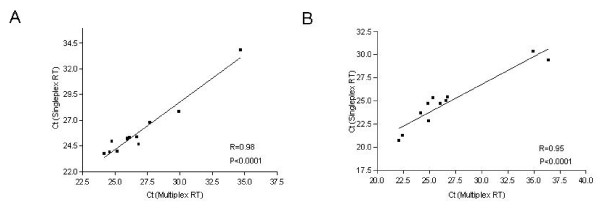
**Correlation scatter plots of Ct values for qPCR using multiplexed or single-plexed RT reactions**. Eleven different miRNA primers were used in single-plex (Y-axis) or 11-plex (X-axis) for reverse transcriptions using A549 (A) and H1299 (B) lung cancer cell lines. The qPCR was done with respected TaqMan probes using the ABI 7900HT instrument.

To evaluate the efficiency of reverse transcription (RT) reactions using different number of primer, we first compared the use of a single miRNA specific primer and an 11 primer-mixed set using 100 ng total RNA isolated from A549 (Figure 1A) and H1299 lung cancer cells (Figure 1B). After the RT reaction, a pre-amplification PCR was performed for 10 cycles and qRT-PCR was carried out using individual TaqMan probes for all 11 genes. Results in Figure 1 showed a strong correlation of Ct values between the two conditions with correlation coefficients at 0.98 and 0.95, respectively, for A549 and H1299 cell lines.

### Reproducibility of Expression Levels between 48.48 Dynamic Arrays and ABI 7900 HT

**Figure 2:**
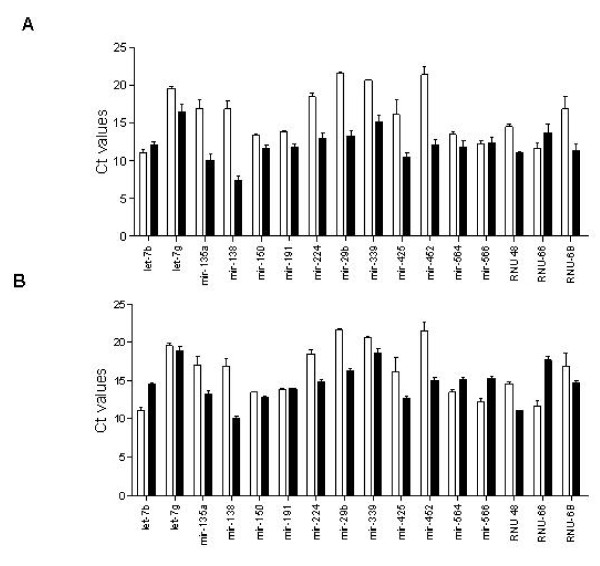
**Ct value comparisons using the 48.48 dynamic array and ABI 7900 HT**. cDNAs were synthesized using 96-plexed primer set and 100 ng total RNA from FF normal lung (A) and FF tumor lung (B) samples. Bars represent the means of Ct values from replicates for the indicated miRNA targets. Open bars: ABI 7900 HT, closed bars: 48.48 dynamic array systems.

As an initial evaluation to determine miRNA expression by using 48.48 dynamic array, we tested its reproducibility by comparing Ct values observed between ABI 7900 HT and Fluidigm dynamic array system using FF samples. Fourteen out of 16 miRNA targets exhibited lower Ct values in the microfluidics 48.48 dynamic array system compared to those obtained by standard qPCR using the ABI 7900 HT (Figure 2A &2B). The mean Ct values between the platforms were 12.48 (± 0.49) for the 48.48 dynamic array and 16.21 (± 0.82) for the ABI 7900 HT (coefficient of variance CV = 0.08 and 0.06, respectively) reflecting a significantly increased sensitivity by the microfluidics array when qPCR reactions are being carried out in nanoliter volumes.

### Comparison of miRNA Expression between FF and FFPE Samples

**Figure 3:**
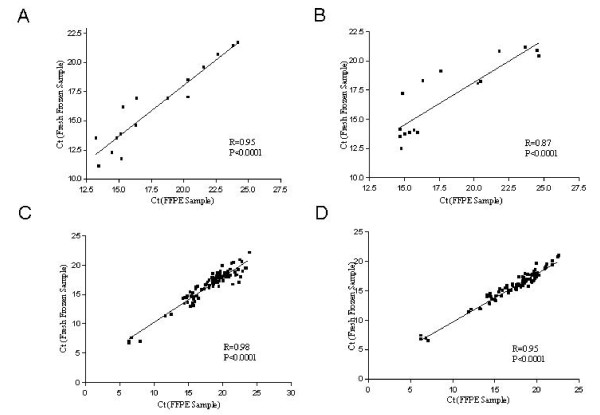
**Correlation between matched FFPE and FF samples in qPCR by ABI vs 48.48 dynamic array platforms**. cDNAs were synthesized using 96-plex primer sets and RNA from both normal lung (A and C) and lung tumor samples (B and D). qPCR reactions were carried out individually for 16 miRNA targets (A and B) by ABI 7900 HT, and for 48 miRNA targets by the 48.48 dynamic array (C and D). Each plot displays mean values calculated from triplicate samples.

We next used both ABI 7900 HT and Fluidigm 48.48 dynamic array to directly compare the qRT-PCR performance using RNA from FF and FFPE samples over a wide range of miRNA gene expression levels. The cDNA was synthesized using a 96-plex primer set and 100 ng of total RNA from both sample types. The Ct values of FFPE samples ranged from 13 to 25 while those of FF samples ranged between 11 to 22 on the standard ABI platform with correlations at R = 0.95 and 0.87, respectively, p < 0.0001) (Figure 3A &3B). When the same reaction were carried out using the 48.48 dynamic arrays, FFPE samples had similarly higher average Ct values compared to FF samples reflecting the generally lower quality of the RNA in FFPE samples (6.84-22.6 in FFPE vs. 6.49-20.97 in FF). Again, the Ct values from both sample types were highly correlated (Figures 3C and 3D).

### Effect of RNA Template Concentration Using 48.48 Dynamic Arrays

**Figure 4:**
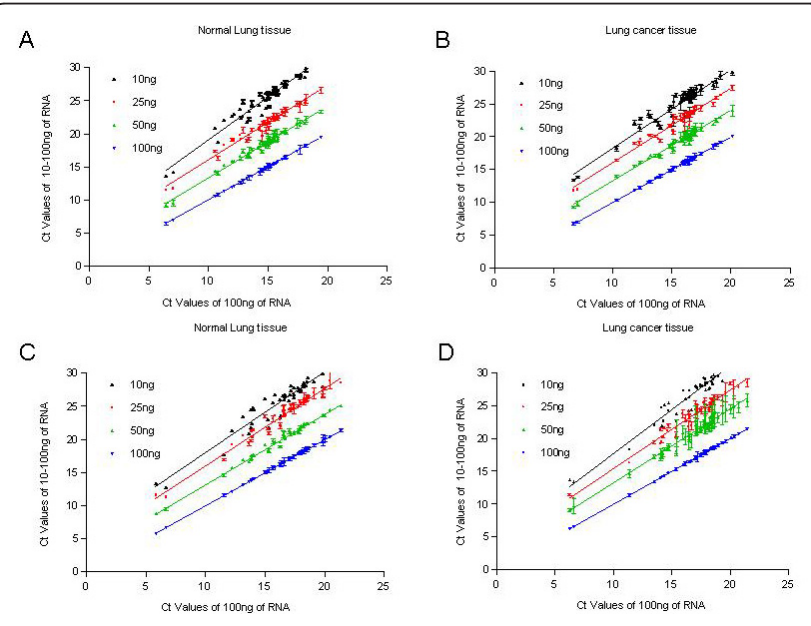
**Effect of in put RNA concentrations on Ct values**. A total of 48 miRNAs were tested using different input amounts of total RNA from FFPE and FF samples for RT and qPCR by the 48.48 dynamic array systems. The Ct values were plotted using the average of the duplicated measurements and the error bar for values on Y-axis. Correlation scatter plots represent the correlation of Ct values for 100 ng RNA (X-axis) and the Ct values for the same FF (A and B) or FFPE (C and D) sample at 10 ng to100 ng concentrations (Y-axis).

To evaluate the dynamic range of the multiplex RT-PCR in the 48.48 dynamic array systems, we evaluated the Ct values for each miRNA target using total input RNA quantities at 10 ng, 25 ng, 50 ng or 100 ng per reaction. The reverse transcription and pre-amplification were carried out in 96-plex format and the qPCR were run using 48.48 dynamic arrays. The scatter plots shown in Figure 4 demonstrates that the correlation coefficients were essentially the same over the tested range of input RNA for both FF (Figure 4A &4B) and FFPE samples (Figure 4C &4D) with the lowest correlation at R = 0.96 (p < 0.0001).

### Comparison of miRNA Expression between the 96.96 Dynamic Array and Affymetrix GeneChip microRNA Microarray

**Figure 5:**
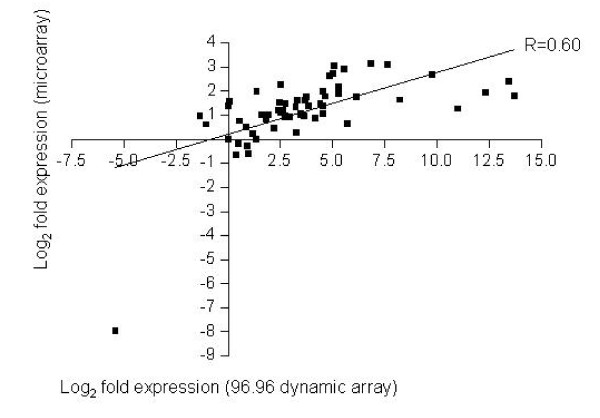
**miRNA expression measurements by Fluidigm dynamic array and Affymetrix microarray**. The raw intensity values of microarray data were transformed to log_2 _values for comparison to PCR Ct values generated by the qPCR platform. Gene expression differences between FF and FFPE were compared against 59 shared genes. Fold differences by the dynamic array (log_2_) were calculated by ΔΔCt method; ΔCt = (target miRNA log_2 _values-hsa-mir-16 log_2 _value), ΔΔCt = (fresh frozen ΔCt-FFPE ΔCt).

We compared miRNA expression levels measured by the 96.96 dynamic array with those obtained from the Affymetrix miRNA GeneChip™. The same samples, FF1 and FFPE9 analyzed by the 96.96 dynamic array were subjected to gene expression profiling using the Affymetrix miRNA arrays. In the Affymetrix miRNA array analysis, 33-35% of miRNA targets were detectable in each sample with a correlation of R = 0.99. Similarly, strong correlations were also obtained using the 96.96 dynamic array when replicate RNA samples were analyzed (R = 0.95). This high correlation value was consistent across both FF and FFPE sample types (Table 1). Overall, the miRNA microarray profiles generated for FFPE and FF samples showed high correlations across all 847 human miRNAs (R = 0.94, p < 0.0001, data not shown). Similarly, the Ct values above detection threshold were obtained for 86 (FF) and 80 (FFPE) of 94 miRNA targets by the qPCR-based analysis using microfluidics. Fifty-nine probes that were called present on Affymetrix miRNA array matched those tested by the 96.96 dynamic array. Comparison of the fold changes between the two samples obtained by Fluidigm and Affymetrix arrays showed an overall correlation of R = 0.60. Significantly, the fold changes detected by Affymetrix microarray ranged between 0 to 3.5, while those by Fluidigm ranged between 0 to 13 reflecting a much higher dynamic range by the microfluidics platform (Figure 5).

**Table 1:** Comparison of gene expression measurements between Affymetrix microarray and Fluidigm 96.96 dynamic array.

Platform	Sample^†^	Background		Signal to Noise	Positive Signal Intensity or Ct			Detection Rate (%)	Replicate Correlation
						
		AVE	MAX		MIN	MAX	AVE		
Microarray (847 miRNA)	FF#1a	2.66	15.79	189	3.49	10530	503	33	0.997
	FF#1b	2.66	11.68	185	1.73	10313	493	33	
	FFPE#9a	2.26	35.38	208	2.34	9331	470	35	0.996
	FFPE#9b	2.22	17.85	209	2.59	8724	464	35	

96.96 dynamic array (94 miRNA)	FF#1a	N/A	N/A	-	7.9	22.4	16.7	91	0.954
	FF#1b	N/A	N/A	-	5.0	22.7	16.2	91	
	FFPE#9a	N/A	N/A	-	5.9	35.9	19.5	85	0.956
	FFPE#9b	N/A	N/A	-	4.2	34.9	19.0	85	

^† ^Samples are as described in the method; N/A, not applicable.

## Discussion

MicroRNAs are potential biomarkers and novel targets for cancer diagnosis, prognosis and recurrence [10,11,21]. Conventional methods for detection and characterization of miRNAs during clinical investigations sometimes fall short because of low throughput, insufficient sensitivity, and relatively high cost. The recently developed Microfluidic technology enables a significantly higher throughput qPCR analysis for a large number of samples; and assays in a much shorter time and at a lower cost compared to the conventional methods [17]. This new real-time PCR technology can be used to perform experimental validation as well as high throughput gene expression measurement with nearly 100-fold less input of sample and reagent [17,19]. In this study, we examined miRNA expression measurements using qPCR-based microfluidic technology using FFPE and matched FF samples. We evaluated this relatively high throughput miRNA profiling method using the standard TaqMan miRNA assays on ABI 7900 HT and the Fluidigm microfluidic dynamic arrays. We also developed and validated miRNA expression assays using cDNA made from either singleplex or multiplex RT reactions and assessed their application for high-throughput miRNA profiling using the microfluidic dynamic arrays.

TaqMan^® ^assays are traditionally used for validation of microarray-based expression analysis [13,22-25]. The RT primer in each TaqMan^® ^miRNA assay is a single-stranded stem-loop RT primer which was developed to allow cDNA detection to be more specific and sensitive as compared to conventional linear primer reverse transcription [22]. Based on this result, Chen, et al. suggested that stem-loop RT primers can be used for multiplex RT reaction and small RNA cloning for better efficiency and specificity [22,24]. In our study, the absolute Ct for miRNA expression levels by singleplex were slightly lower than those obtained in multiplex conditions, but both methods showed high correlation compared to results of the qRT-PCR (A549 cells, R = 0.98 and H1299 cells, R = 0.95; p < 0.0001) (Figure 1).

Several studies have demonstrated that FFPE samples can be used for miRNA profiling analysis [13,16]. However, most of the studies are based on microarray data and validated using low throughput Applied Biosystems qPCR platforms [13,15,16]. Here, we compared the correlation of miRNA expression profiles between FFPE and matched FF samples using both qPCR and array bases platforms. We observed a high correlation of miRNA expression levels (R = 0.95 and R = 0.98; p < 0.0001) measured for both sample types using the ABI 7900 HT and the 48.48 dynamic array (Figure 3). FF samples appeared to contain higher levels of miRNAs than FFPE samples in our study, consistent with results observed by Leite, et al [13] using standard qPCR assays. This could be due to the loss of miRNA during paraffin embedding process or RNA extraction. Considering the minimal influence on miRNA measurements, the small difference of Ct values between FFPE and FF samples is not expected to affect the result of the study, particularly for Fluidigm based studies since the reference miRNA targets are measured on the same array for the exact same sample.

To compare between qPCR platforms, we investigated 16 different miRNA targets using both ABI 7900 HT and Microfluidic technology with FF RNA samples. The Ct value of the 48.48 dynamic array system shifted toward lower Ct values compared to those observed by ABI 7900 HT in a 5 μl reaction. In our hands, the mean Ct value difference was 3.79 between the two systems and the coefficient of variation across the 128 reactions in the 48.48 dynamic array system was 8.9% (Figure 2). In addition, we showed that as little as 10 ng of total RNA could be used to detect all 48 miRNAs with 96-multiplexing RT reaction in both FFPE and FF samples (Figure 4). The minimal correlation coefficient observed was 0.96 for 10 ng vs.100 ng input RNA for both FF and FFPE samples (P < 0.0001). Lao et al. [24] suggested that 10 ng of a human lung sample can be assessed with a substantial degree of accuracy without statistical variations from stochastic effects when multiplex RT reactions are employed. Therefore, our data are consistent with those previously reported and indicates that 96-multiplexed miRNA RT reactions can provide reliable miRNA profiles when using low input amounts in the dynamic array systems. Although just a few samples were used in this study, the robustness of the assay was demonstrated consistently for all tested assays varying at a wide range of Ct values (Figures 3 and 4).

To determine the concordance between qPCR-arrays and microarrays, we compared miRNA expression using 96.96 dynamic arrays and the hybridization-based miRNA array offered by Affymetrix. We used the same samples for both analyses and the overall correlation for the 59 shared genes was R = 0.60 (p < 0.0001) for miRNA expression between the two platforms. This moderate correlation likely reflects the use of fundamentally different principles for gene expression measurements in these two platforms. Fluidigm uses the quantitative PCR assays which are highly sensitive with a dynamic range of at least 6-7 logs [19,22]. In contrast, Affymetrix GeneChip is primarily based on hybridization of the labeled probes to the matching oligonucleotides that are affixed to a matrix. The dynamic range of the microarray is usually 3 to 4 logs [25,26]. In our hands, the maximum fold change observed was around 3 for Affymetrix arrays and 13 for Fluidigm dynamic array (Figure 5).

Several studies have previously reported a similar rate of inter-platform concordance among different miRNA microarrays and the different expression values from each miRNA microarray platform when compared to qPCR values [27-29]. Although highly sensitive, the quality of the gene expression assessment by the TaqMan-based method is dependent largely on the specificity of the probe to discriminate among highly conserved miRNA target sequences as well as the sensitivity of the assay probes to quantitatively measure the target miRNA over a wide range of expression levels. The small 18-25 nucleotide length of the miRNA targets creates a challenge to meet these requirements. Pradervand, et al. [27] observed that the different GC content of mature miRNA sequences contributes to higher difference values between both Affymetrix and qPCR. This same study also showed a lower correlation between qPCR and Affymetrix while reporting a higher correlation between qPCR based assay and other platforms (R = 0.8-0.9). In the study by Chen, et al. [29], a correlation of 0.44 (p < 0.0001, N = 84) was reported between TaqMan qPCR-array and microarray. The variation observed was thought to result from the low abundance miRNAs reflecting the different sensitivities of the two platforms. Therefore, the assessment of miRNA expression in a given system should be interpreted with caution and it requires validation using at least two different platforms when the correlation is poor or the expression level of the target is low.

## Conclusion

We demonstrated that multiplexing RT reactions with stem-loop primers can be adapted with relative ease to a new qPCR-array based microfluidic platform to profile miRNA expression profiling. We showed that this approach is highly reproducible and correlates closely with standard ABI7900 systems yet offers higher throughput, with much lower sample input, and reagent usage. We believe that the microfluidic dynamic array technology could be used to develop cost effective and customized assays with rapid turn-around for profiling and validating of miRNA expression.

## Methods

### RNA extraction from FFPE, FF samples and lung cancer cell lines

FF and corresponding FFPE samples were obtained from lung cancer (FF1 and FFPE9) or non-diseased lung tissues (FF4 and FFPE7) that had been preserved between 2007 and 2008 following the approved Mayo Clinic Institutional Review Board protocol. FFPE samples were cut to 10 μm thickness and several tissue slices were put into a 1.5 ml tube. One milliliter of xylene was added for deparaffinization followed by mixing twice with a high speed vortex for 3 min at room temperature. Total RNA was then extracted with the Qiagen miRNeasy FFPE kit (Valencia, CA) and/or RecoverAll (Ambion Inc. Austin, TX) following manufacturers' protocols. Fresh-frozen tissues were extracted using Qiagen miRNeasy kit (Valencia, CA) following manufacturer's protocols. The isolation procedure for FF and FFPE samples were performed in duplicate to derive samples FF1a and FF1b, and FFPE9a and FFPE9b. Human lung cancer cell lines H1299 and A549 were cultured in RPMI 1640 growth media with 10% fetal bovine serum and 1% penicillin (50 IU/mL) and streptomycin (50 μg/ml). Total RNA was isolated from TRIzol (Invitrogen, Carlsbad, CA). RNA quantity was determined using Nanodrop (Thermo Scientific, Waltham, MA) and the quality was assessed by Agilent 2100 Bioanalyzer (Agilent Technologies, Santa Clara, CA).

### MicroRNA reverse transcription

The 15 μl reverse transcription reaction contained 2 μl of either 5, 12.5, 25 or 50 ng/μl of total RNA, 0.2 μl of 100 nM dNTP, 0.2 μl of RNase inhibitor 20 U/μl, 1.5 μl of reverse transcriptase (50 U/μl), 8 μl of 11-or 96-plex reverse primer (mixed to allow a final concentration of 0.05X of each) and 1.6 μl of H_2_O. 2 μl of 5 ng of total RNA and 3 μl of 5X reverse primer were used in the single-plex RT reaction. All reagents were purchased from Applied Biosystems, Inc. (Foster City, CA). The reaction mixture was mixed with RNA and incubated as follows; 16°C for 30 min, 42°C for 30 min and then 85°C for 5 min. A list of all 96 tested assays is available upon request. The 11 primer set that was used for the RT reaction included: RNU66, RNU6B, mir-135a, mir-564, mir-29b, mir-339, mir-138, mir-425, mir-191, let-7 g and mir-566 (Applied Biosytems, Foster City, CA).

### Pre-PCR amplification

For pre-amplification of cDNA, we pooled 11- or 96 TaqMan Assays at a final concentration of 0.2X for each assay. Pre-PCR amplification reaction was done at 10 μl containing 5 μl TaqMan PreAmp Master Mix (2X), 2.5 μl of 11- or 96-pooled TaqMan assay mix (0.2X) and 2.5 μl of cDNA. The pre-amplification PCR performed at one cycle 95°C for 10 min, 10 cycles at 95°C for 15 sec and then 60°C for 4 min. After pre-amplification PCR, the product was diluted 1:5 with dH_2_O and stored at -80°C until needed.

### Real-time qPCR

qRT-PCR was carried out using ABI 7900 HT Real-time PCR system in a 384 well plate format. PCR reaction of 5 μl contained 2.5 μl of TaqMan PCR Master Mix-UNG (2X), 0.25 μl of each TaqMan assay probe (20X), 1.25 μl of diluted cDNA and 1 μl of H_2_O. The PCR was performed at 95°C for 10 min, followed by 40 cycles at 95°C for 15 sec and 60°C for 1 min. The data was analyzed with ABI RQ Manager software (Foster City, CA) after exportation as a SDS file.

### miRNA expression analysis using 48.48 and 96.96 dynamic array

Reverse transcription was carried out as described above using pooled miRNA primers with 10 ng, 25 ng, 50 ng and 100 ng of total input RNA. Pre-amplification was performed with a 96 pooled (final 0.2X of each) TaqMan assay. After pre-amplification PCR, the product was diluted 1:5 with dH_2_O and stored in -80°C until needed. qPCR was carried out using the 48.48 or 96.96 dynamic array (Fluidigm Corporation, CA, USA) following the manufacturer's protocol [19]. Specifically, a 5 μl sample mixture was prepared for each sample containing 1 × TaqMan Universal Master Mix (No UNG), 1 × GE Sample Loading Reagent (Fluidigm PN 85000746) and each of diluted pre-amplified cDNA. 5 μl of Assay mix was prepared with 1 × each of TaqMan miRNA assay and 1 × Assay Loading Reagent (Fluidigm PN 85000736). An IFC controller was used to prime the fluidics array (chip) with control line fluid and then with samples and assay mixes in the appropriate inlets. After loading, the chip was placed in the BioMark Instrument for PCR at 95°C for 10 min, followed by 40 cycles at 95°C for 15 sec and 60°C for 1 min. The data was analyzed with Real-Time PCR Analysis Software in the BIOMARK instrument (Fluidigm Corporation, CA, USA).

### miRNA microarray

The miRNA microarray profiling was performed using Affymetrix GeneChip miRNA arrays (Santa Clara, CA, USA) according to manufacturer's recommended protocol. Briefly, 1 μg of total RNA was labeled by polyA polymerase addition using the Genisphere FlashTag HSR kit following the manufacturer's recommendations (Genisphere, Hatfield, PA). RNA was hybridized to the Affymetrix miRNA array as recommended by the vendor. Standard Affymetrix array cassette staining, washing and scanning was performed using the post-hybridization kit (#900720; Affymetrix) and GeneChip Scanner 3000. Feature extraction was performed using Affymetrix Command Console software. The raw data were treated by the following workflow: background detection, RMA global background correlation, quantile normalization, median polish and log2-transformed with miRNA QC tool software (Affymetrix).

### Statistical Analysis

Statistical Analysis was performed using GraphPad Prism 4 (GraphPad Software, Inc.). The Pearson correlation coefficient(R) was employed to determine the correlation of efficiency of RT reaction and expression of FFPE and FF samples.

## Authors' contributions

JSJ performed the research, analyzed the data and wrote the paper. VS, RMF, FR and DS participated in the Fluidigm and/or microarray experiments. MZ performed RNA preparation and WL participated in study discussions. JJ directed research and analyzed the data. RMF, CPK and JJ revised the manuscript. All authors read, corrected and approved the final manuscript.
